# Supplementary material for: Telemonitoring for perioperative care of outpatient bariatric surgery: Preference-based randomized clinical trial
Source: PLoS One. 2023 Feb 22;18(2):e0281992. doi: 10.1371/journal.pone.0281992 (PMC9946229; doi:10.1371/journal.pone.0281992)
Supplement: S2 File — (PDF) [file pone.0281992.s003.pdf]

Clinical Investigation Plan

Postbariatric EArly discharge Controlled by Healthdot (PEACH)

This Clinical Study* protocol template is for use with ICBE studies formerly known as H, H-Lite, J, J-Lite. These studies undergo full ICBE Board review (not offline review). This template is ISO 14155 compliant; however, not all clinical studies will follow ISO 14155 and thus non-applicable elements can be noted as such.

*A systematic investigation in one or more human subjects, undertaken to assess the safety or performance of a medical device (ISO 14155). NOTES: 1. "Clinical trial" and "clinical study" are synonymous with "clinical investigation"; 2. The Netherlands *WMO* defines a "clinical trial" as medical research and in which persons are subjected to treatment or are required to follow a certain behavioral strategy.

You must undergo a Regulatory Intake with the Director of Q&R before you submit your study to ICBE for review. This ensures your study considers potentially relevant matters such as REC review, database registration, prototype release/DOC, competent authority notification, etc.

**Document history**			
Version:	Date:	Author:	Summary of changes
0.1	*2020-JAN-14*	*Simone Ordelman*	*First draft*
0.2	*2020-JAN-31*	*Joerg Liebmann*	*Filled in some details, updated device description*
0.3	*2020-FEB-04*	*Simone Ordelman*	Implemented changes after discussion with clinical site
0.4	2020-FEB-11	Joerg Liebmann	Implemented changes after discussion with clinical site
0.5	2020-FEB-24	Simone Ordelman	Implemented feedback from clinical site received via e-mail
0.6	2020-APR-20	Simone Ordelman	Implemented feedback from clinical site received via e-mail
0.7	2020-APR-30	Simone Ordelman	Implemented feedback from clinical site
0.8	2020-JUN-02	Joerg Liebmann	Added risk analysis

0.9	2020-JUN-08	Joerg Liebmann/Simone Ordelman	Adressed Coach comments
0.91	2020-JUN-16	Joerg Liebmann	Adressed secretary comments
0.92	2020-JUN-24	Joerg Liebmann	Adressed reviewer comments
0.93	2020-JUN-29	Joerg Liebmann	Updated link to other studies and reasoning for design.
1.0	2020-JUL-24	Joerg Liebmann	Approved by ICBE
1.1	2020-NOV-05	Joerg Liebmann	Changes made upon request of METC
2.0	2020-NOV-12	Joerg Liebmann	Approved by ICBE
2.1	2020-DEC-02	Joerg Liebmann	Changes made upon request of METC
3.0	2020-DEC-08	Liesbeth Vonk	Approved by ICBE

APPROVAL / PROTOCOL SIGNATURE SHEET

**Protocol Title :**	Postbariatric EArly discharge Controlled by Healthdot (PEACH)
**Protocol Number :**	ICBE-2-36455
**Protocol Version :**	3.0
**Protocol Date**	2020-DEC-08
**Sponsor :**	Philips Electronics Nederland BV, acting through Research, Eindhoven, NL

**Regulatory Representative of Sponsor**<br>Senior Manager Regulatory Affairs at Philips Innovation & Strategy	____________________<br>Name	____________________<br>Signature
**or**		____________________<br>Date (dd-MMM-YYYY)
**Head of Department of Sponsor**		

By signing below, I the investigator, agree to conduct the study in accordance with the study protocol as outlined in this document.

I will ensure that the investigation materials supplied by the study sponsor will be used as specified in the study protocol and in the User's Manual for the specified device used in the study protocol.

**Principal Investigator**	____________________<br>Name	____________________<br>Signature
		____________________<br>Date (dd-MMM-YYYY)

ROLES / RESPONSIBILITIES

This section contains a list of all parties and persons involved in the Clinical Investigation.

**Coordinating investigator/ Project leader**	Not applicable
**Principal investigator(s)**	Simon Nienhuijs General Surgeon, specialised in Bariatric Surgery at Obesitas center, Catharina Hospital, Eindhoven<br>simon.nienhuijs@catharinaziekenhuis.nl
**Investigational Site**	Catharina Hospital, Michelangelolaan 2, 5623 EJ Eindhoven, The Netherlands
**Other participating sites**	Not applicable
**Sponsor (the individual or organization taking responsibility and liability for the initiation or implementation of a clinical investigation)**	Philips Electronics Nederland BV, acting through Research, Eindhoven, NL
**Subsidizing party**	Not applicable
**Independent physician(s) (the Netherlands only)****	Evert Koldewijn, Urologist at Urology Department, Catharina Hospital, Eindhoven
**Laboratory sites**	Not applicable
**Other / Appendix**	
Arthur Bouwman<br>Catharina Hospital<br>arthur.bouwman@catharinaziekenhuis.nl<br><br>Jai Scheerhoorn<br>Catharina Hospital<br>jai.scheerhoorn@catharinaziekenhuis.nl<br><br>Joerg Liebmann<br>Philips Research<br>joerg.liebmann@philips.com<br><br>Liesbeth Vonk<br>Philips Experience Design<br>liebeth.vonk@philips.com	

Gewijzigde v

TABLE OF CONTENTS

APPROVAL / PROTOCOL SIGNATURE SHEET 3

ROLES / RESPONSIBILITIES 4

LIST OF ABBREVIATIONS AND RELEVANT DEFINITIONS 6

1 EXECUTIVE SUMMARY 8

2 INVESTIGATIONAL DEVICE 13

2.1 Device Summary 13

2.2 Intended purpose 16

2.3 Device Description 17

3 JUSTIFICATION 18

4 RISKS AND BENEFITS ASSESSMENT 19

5 OBJECTIVES AND HYPOTHESES 20

6 CLINICAL INVESTIGATION DESIGN 22

6. 1 General 22

6.2 Investigational device(s) and comparator(s) 23

6.3 Subjects 23

6.4 Procedures 25

6.5 Monitoring plan 32

7 STATISTICAL CONSIDERATIONS 32

8 DATA MANAGEMENT 34

9 AMENDMENTS 35

10 DEVIATIONS 35

11 DEVICE ACCOUNTABILITY 35

12 STATEMENTS OF COMPLIANCE 36

13 INFORMED CONSENT PROCESS 37

13.1 Consent Process 37

13.2 New Information about the Study 38

14 ADVERSE EVENTS AND DEVICE DEFICIENCIES 38

15 INVESTIGATOR BROCHURE (IB) 42

16 VULNERABLE POPULATION 42

17 SUSPENSION OR PREMATURE TERMINATION 43

18 PUBLICATION POLICY 44
19 BIBLIOGRAPHY 44

Gewijzigde v
Gewijzigde v

LIST OF ABBREVIATIONS AND RELEVANT DEFINITIONS

ABR	ABR form, General Assessment and Registration form, is the application form required for submission to the accredited Ethics Committee<br>*In Dutch, ABR = Algemene Beoordeling en Registratie*
ADE	Adverse Device Effect<br>Adverse event related to the use of an investigational medical device
AE	Adverse Event<br>Any untoward medical occurrence, unintended disease or injury, or untoward clinical signs (including abnormal laboratory findings) in subjects, users or other persons, whether or not related to the investigational medical device
AR	Adverse Reaction
CA	Competent Authority
CCMO	Central Committee on Research Involving Human Subjects<br>*In Dutch, CCMO = Centrale Commissie Mensgebonden Onderzoek*
Clinical Investigation	Systematic investigation in one or more subjects, undertaken to assess the safety and performance of a medical device
CIP	Clinical Investigation Plan<br>Document that state(s) the rationale, objectives, design and proposed analysis, methodology, monitoring, conduct and record-keeping of the clinical investigation<br>NOTE: The term “protocol” is synonymous with “CIP”. However, protocol has many different meanings, some not related to clinical investigation, and these can differ from country to country.
CRF	Case Report Form<br>Set of printed, optical or electronic documents for each subject on which information to be reported to the sponsor is recorded, as required by the CIP
CV	Curriculum Vitae
DSMB	Data Safety Monitoring Board
EC	Ethics Committee
ECG	Electrocardiogram
Endpoint	Principal indicator(s) used for assessing the primary hypothesis of a clinical investigation
ERABS	Enhanced Recovery After Bariatric Surgery
EU	European Union
EWS	Early Warning Score
GCP	Good Clinical Practice
IB	Investigator’s Brochure
IC	Informed Consent<br>The informed consent is documented by means of a written, signed and dated informed consent form.<br>The informed consent process is the process by which an individual is provided information and is asked to voluntarily participate in a clinical investigation.
Investigation site	Institution or site where the clinical investigation is carried out
IRB	Institutional Review Board
Hypothesis	Testable statement, resulting from the objective, regarding the investigational medical device safety or performance used to design the clinical investigation and that can be accepted or rejected based on results of the clinical investigation and statistical calculations.<br>NOTE: The primary hypothesis is the determinant of the investigational medical device safety or performance parameters and is usually used to calculate the sample size. Secondary hypotheses concerning other points of interest can also be evaluated.

Investigator	Individual member of the investigation site team designated and supervised by the principal investigator at an investigation site to perform critical clinical-investigation-related procedures or to make important clinical investigation- related decisions.<br>NOTE: An individual member of the investigation site team can also be called “sub-investigator” or “co-investigator”.
LOS	Length of hospital stay
METC	Medical Research Ethics Committee (MREC)<br>*In Dutch, METC = Medisch Ethische Toetsing Commissie*
Objective	Main purpose for conducting the clinical investigation
Point of enrollment	Time at which, following recruitment, a subject signs and dates the informed consent form
POD	postoperative day
SADE	Serious Adverse Device Effect<br>Adverse device effect that has resulted in any of the consequences characteristic of a serious adverse event or that might have led to any of these consequences if suitable action had not been taken or intervention had not been made or if circumstances had been less
SAE	Serious Adverse Event<br>Adverse event that<br>a) led to death,<br>b) led to serious deterioration in the health of the subject, that either resulted in<br>1) a life-threatening illness or injury, or<br>2) a permanent impairment of a body structure or a body function, or<br>3) in-patient or prolonged hospitalization, or<br>4) medical or surgical intervention to prevent life-threatening illness or injury or permanent impairment to a body structure or a body function,<br>c) led to foetal distress, foetal death or a congenital abnormality or birth defect<br>NOTE Planned hospitalization for a pre-existing condition, or a procedure required by the CIP, without serious deterioration in health, is not considered a serious adverse event.
Sponsor	Individual or organization taking responsibility and liability for the initiation or implementation of a clinical investigation
UADE	Unanticipated Adverse Device Effect<br>Serious adverse device effect which by its nature, incidence, severity or outcome has not been identified in the current version of the risk analysis report
WBP	Personal Data Protection Act (in Dutch: Wet Bescherming Persoonsgevens)
WMO	Medical Research Involving Human Subjects Act<br>*In Dutch, WMO = Wet Medisch-wetenschappelijk Onderzoek met Mensen*

1 EXECUTIVE SUMMARY

**Study Title & ID**	PJ-017309 Postbariatric EArly discharge Controlled by Healthdot (PEACH)
**Short Study Title**	PEACH
**Project Name**	[Acute Care Solutions]()
**Brief Summary of Study**<br>***Provide a lay-language abstract of your study plan (100-300 words)***<br>*https://humansubjects.stanford.edu/new/docs/glossary_definitions/lay_language.pdf*	This clinical investigation is a single center patient preference trial in a tertiary hospital in the Netherlands, designed to compare the outcome of two different recovery paths after standard of care bariatric surgery. The difference between both recovery paths is that half of the patients will get the standard of care by staying one night in the hospital before returning home (group B), while the other half will receive a Healthdot directly after surgery and leave the hospital on the same day (evening) (group A). 200 patients will be recruited and can choose whether they want to be assigned to the the regular recovery path or receive a Healthdot and leave the hospital on the same day. If they have no preference they will be randomly assigned to oe of the two groups. Patients in the outpatient recovery group will wear the Healthdot for 7 days at home and vital signs (heart rate and respiratory rate, together with context data on activity and posture) will be transmitted to the hospital to monitor recovery. The study is mainly designed to investigate if the clinical outcome in both groups is equal (non-inferiority) based on a combined outcome measures like 30 days readmission rate and patient satisfaction.
**Key Words Associated with Study**<br>**Provide 3-5 PubMed MeSH keywords**<br>**(https://www.ncbi.nlm.nih.gov/mesh)**	bariatric surgery, outpatients, telemedicine
**Country(s) from where the data originates (where collected)**	☒ Netherlands ☐ USA ☐ Germany ☐ China<br>☐ India ☐ Belgium ☐ UK ☐ Kenya<br>☐ Other:
**ISO 14155 compliance**	☒ This study will be conducted per the requirements of ISO 14155

Gewijzigde v

Gewijzigde v

Gewijzigde v

	☐ This study WILL NOT be conducted per the requirements of ISO 14155. For example studies formerly known as H-lite and J-Lite
**Is this study part of a student project/thesis?**	☒ No<br>☐ Yes, Student Name: University:
**Primary objective (only one)**	To evaluate whether performing outpatient recovery after standard bariatric surgery is not inferior to the current recovery path based on a combined outcome measure.
**Secondary objective(s)**	• To assess Patient satisfaction (on a scale of 1-10) in both groups<br>• To assess feasibility of outpatient recovery after standard bariatric surgery by evaluating recruitment rate, adherence to protocol and randomization, and the amount of missing data.<br>• To evaluate the percentage and total number of false-positive notifications from the Healthdot system.<br>• To evaluate the percentage of missed events (i.e. false negatives) from the Healthdot system.<br>• To evaluate the total number of missed minor events from the Healthdot system.<br>• To compare the clinical decisions made on basis of remote monitoring with the Healthdot system, to decisions which also include information from a telephone consultation.<br>• To evaluate the outcome of patients who choose HD versus patients who were randomized to HD on a combined outcome measure as defiend for the primary endpoint.<br>• To compare the number of adverse events in both groups<br>• To compare the use of pain medication during day of surgery until evening in both groups.<br>• To evaluate the impact of outpatient recovery and the Healthdot on patient and health professional satisfaction<br>• To evaluate the costs involved with outpatient recovery after standard bariatric surgery supported by Healthdot.<br>• To evaluate usability of the visualization of Healthdot data on the Guardian dashboard.
**Participating parties and their roles**	• Catherina Hospital: study site, executing study<br>• Philips Research:, scientific support<br>• Philips Electronics Nederland B.V., CTO Ventures: Provide Healthdot system
**Subjects**	In this clinical investigation, 200 patients will be enrolled. Patients included in the study can choose if they want to be assigned to the regular care path or receive a Healthdot and leave the hospital on the same day of surgery. If the patient has no preference, he/she is randomly assigned to either recovery path.

	**Inclusion criteria for subject selection**<br>- Adult (equal or greater than 18)<br>- Approval for primary bariatric surgery (gastric sleeve or bypass) by a multidisciplinary bariatric team<br>- Willing and able to sign informed consent form<br>- Able to understand instructions<br>- In possession of a telephone on which patient can be reached for the duration of participation (day 1-8)<br>- An adult person must be present at the same location as the patient during the first night following surgery who is able to mobilize help or seek medical care if necessary.<br><br>**Exclusion criteria for subject selection**<br>- Patients of psychiatric wards, inmates of prisons, or other state institutions<br>- Investigator or any other team member involved directly or indirectly in the conduct of the clinical study<br>- Any skin condition, for example prior rash, discoloration, scars or open wounds at the area (lower left rib) where the Healthdot needs to be placed<br>- Known allergy for the tissue adhesive used in the Healthdot (white band-aid)<br>- Use of topical that is known to influence the skin at the test area (such as medical and non-medical creams or lotions)<br>- Patient with active implantables such as Implantable Cardioverter Defibrilator (ICD) and pacemaker<br>- Expected participation less than 8 days<br>- Left lower rib (place where Healthdot will be applied) is involved in the area of surgery, area of disinfection or area where bandages are needed.<br>- Patients with antibiotic resitant infections (e.g. MRSA)
**Investigation design**	This clinical investigation is designed as a single center patient preference non-inferiority trial. (Randomized when patient doesn't have a preference).
**Investigation procedures**	The first subject is expected to be enrolled in December 2020. The Clinical Investigation is expected to take 8 months. The duration of the active participation of each subject is 8 days. The patients assigned to the outpatient recovery group wearing the Healthdot will get the Healthdot applied after surgery and leave the hospital on the same day. They will be expected to wear the Healthdot for 8 days overall. In total, 200 patients will be enrolled. The enrolment period will be 6-7 months. Half of the patients will be assigned to the outpatient recovery group, and half of the patients will be assigned to the regular recovery group (standard

	of care workflow). Patients included in the study can choose in which group they want to be. This way they can choose whether they receive outpatient recovery or the regular recovery path (staying one night in hospital). If the preferred group is full, the patient can choose to participate in the other group, or to withdraw from the study. If the patient has no preference, he/she is randomly assigned to either recovery group, or if one group is full, to the group for which 100 patients have not yet been included. Patient preference is recorded in the eCRF and analysed.
**At-Home Subject Procedures**	☐ NOT APPLICABLE<br>At home, the subjects are expected to wear the Healthdot device for seven days and interact with their caregivers as needed. Patients are expected to fill in a questionnaire at the end of the 7 days at home.
**Device(s) / Other support (s)**<br><br>**Check each box as appropriate and add a brief description of the device(s)—e.g., fitness tracker, VR goggles, breast pump, electric toothbrush, ELAN, MRI scanner.**<br><br>**Device(s) and/or other support(s) will be described fully in Section 2.1 of the study protocol.**	☐ Mock-up (see ICBE FAQ definition):<br>☒ Survey/questionnaire (if copyrighted, ensure permission for use): Satisfaction questionnaire to patient and physician.<br>☐ Interview/focus group with audio/video taping**:<br>☐ Interview/focus group without audio/video taping**:<br>**see decision flowchart regarding interviews vs QI vs consulting https://share-intra.philips.com/sites/STS20131115093003/ICBE-FAQ/Lists/Photos/interviews%20criteria%20flowchart%20feb2018.pptx<br>☒ FDA or CE-approved medical device within intended use: Philips Intellivue Guardian system already installed at hospital to view data from Healthdot wearable.<br>☐ FDA or CE-approved medical device outside intended use:<br>☒ FDA or CE-approved non-medical device within intended use: Gateway for LoRa reception inside hospital<br>☐ FDA or CE-approved non-medical device outside intended use:<br>☐ In vitro medical device:<br>☒ Medical device prototype***: Healthdot<br>☐ Non-medical device prototype***:<br>☐ Software prototype***, non-medical:<br>☐ Software prototype***, medical:<br>☐ Other:<br>☐ None<br><br>***Before a study employing prototypes may start, a Declaration of Conformity (DoC) needs to be issued by the Director Regulatory Affairs. With this DoC, Philips declares that the

Gewijzigde v

	device can safely be used in the study. It may be needed to work according to some formal standards to build up this evidence (e.g. with respect to bio-compatibility or sterility).<br><br>*** ☒ DoC needed (brief overview of the actions that will be taken to get the Declaration of Conformity. See Philips Research QMS procedure “Procedure Product Release (QR-PRO-36”. Give details of how the project will deal with the regulatory requirements. Consult the Q&R office when needed): A declaration of conformity will be requested from regulatory affairs based on the Design history file and the Investigational Medical device Dossier of the Healthdot.
**Milestones**	Study start (first patient first visit): Dec 2020 (planned)<br>Interim analysis (after 50 patients): Mar 2021 (planned)<br>Study end (last patient last visit): Jul 2021 (planned)<br>Final Report: Oct 2021 (planned)
**Claims**	Will you obtain regulatory (FDA, CE) approval for a new product or service, a new indication, or a new marketing claim?<br>☒ Yes ☐ No<br><br>If YES, in what countries will the claims be registered?<br>☒ Netherlands ☐ USA ☐ Germany ☐ China<br>☐ India ☐ Belgium ☐ UK ☐ Kenya<br>☐ Other:<br><br>Have you developed a list of claims that you would like to assert for your product or service? ☐ Yes ☒ No
**External Medical Ethics Committee Approval**<br><br>**Note: if your document is not in English or Dutch, include an English translation**	☐ Not Required (not a local/state/country requirement)<br>☒ Required (apply for it AFTER ICBE approval)<br>☐ Approval already obtained (upload for review by ICBE)<br>☐ REC Review is waived (upload review waiver)<br>☐ REC has waived requirement for informed consent (upload waiver)<br>☐ Check this box if more than one REC is involved in this study and identify them:
**Submission to Competent Authorities**	☐ Not applicable<br><br>Check the applicable option concerning Competent Authority approval for medical device studies:<br>☐ No submission to Competent Authority needed, since no non-released medical device involved<br>☒ Approval from Competent Authority is mandatory for this study with a non-released medical device

	Note: Released medical device means a device that is e.g. CE-marked (93/42/EEC) if study takes place in EU, or has 510k if study takes place in USA.
**Database registration**	☐ No<br>☒ Yes : ClinicalTrials.gov
**Type of Legal Agreement for this study**	☐ None required ☒ MRA Exhibit<br>☐ Contract ☐ Purchase Order
**Status of Legal Agreement**	☐ NA ☐ Not started ☒ In Process ☐ In Place<br>Name of Legal Representative: Godber, Nicole
**IP Strategy**	IP rights of Philips in this project are described in the eMTIC contract and the appended Exhibit specific for this study.
**ICBE Training is required for all key study personnel**	☒ YES, this training is completed as required by ICBE*<br>☐ NO, this training is not completed as required by ICBE*:<br>*See FAQ: https://share-intra.philips.com/sites/STS20131115093003/ICBE-FAQ/Lists/Posts/Post.aspx?ID=159

Gewijzigde v

2 INVESTIGATIONAL DEVICE

2.1 Device Summary

This chapter contains a summary description of the **investigational device** and its intended purpose.

**Summary device description**	The Healthdot device is a body worn (chest) accelerometer based measurement device, suitable for 24/7 data collection. The device is intended to be worn attached to the skin by means of a skin adhesive patch, typically placed on the left lower rib. The general operating principle is based on continuous collection and periodic transmission of heart rate, respiratory rate, activity and posture by means of processing of accelerometer signals. These values are transferred from the Healthdot device via an RF signal to a receiver, which transfers the data to a backend and makes the data available for viewing on a (online) dashboard. The transmit interval is typically once every 5 to 15 mins.
**Summary intended purpose**	The Healthdot wearable and backend is indicated for use by health care professionals in combination with a clinical information management system whenever there is a need for acquisition of the patient parameters respiratory rate and heart rate.
**Population description**	Patients approved for primary bariatric surgery (gastric sleeve or bypass) by a multidisciplinary bariatric team
**Manufacturer**	Philips Electronics Nederland B.V., CTO Ventures, Eindhoven, NL
**Device model/type**	Healthdot 3 (also called HealthdotC)
**Software version**	Healthdot device: Atlas algorithm version D.10

	Philips Intellivue Guardian: release E2, which uses Philips Intellibridge Enterprise release 13.1, CE marked.
**Accessories**	Not applicable
**Traceability procedure**	The device traceability during and after the clinical investigation is documented in form “Device Accountability”. This record is intended to document the physical location of all investigational devices from shipment of investigational devices to the investigation sites until return or disposal.<br><br>The device is tracked to and from the investigational site by the Study Manager, while the trace to the subjects is done by the Principal Investigator. At clinical investigation termination or closure, device retrieval or disposure will be checked by the Monitor.<br><br>The devices are uniquely identified by a label containing the serial numbers.
**Required training**	The investigators will be instructed how to handle the Healthdot and where to position it. This will also be described in an instruction for use provided to the investigator. No specific training is required. The patient will be informed on how to act and what to avoid while wearing the device with a separate Patient card.
**Specific procedure acts**	Not applicable

**Device Labeling**	The HealthdotC contains labeling on the package which is listed and described below. The HealthdotC also has an IfU which will be handed over to the investigators. The device label itself is shown in Figure 1 below. Additionally the device will be labled with:

"Uitsluitend voor klinisch onderzoek"

- Explanation of symbols

Symbol	Description
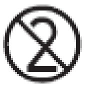	Do Not Re-use
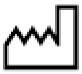	Year of manufacture
LOT	Lot number
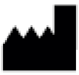	Manufacturer
REF	Batch Code
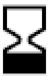	Use By Date
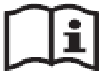	Read Instructions for Use
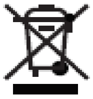	Not for general waste.
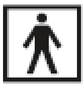	Type BF applied part
IP55	Ingress protection
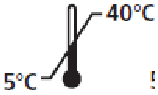	Temperature limitation
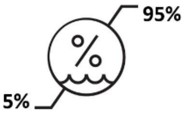	Humidity limitation
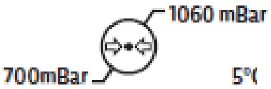	Pressure limitation

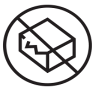	Do not use if package is broken
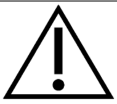	Caution

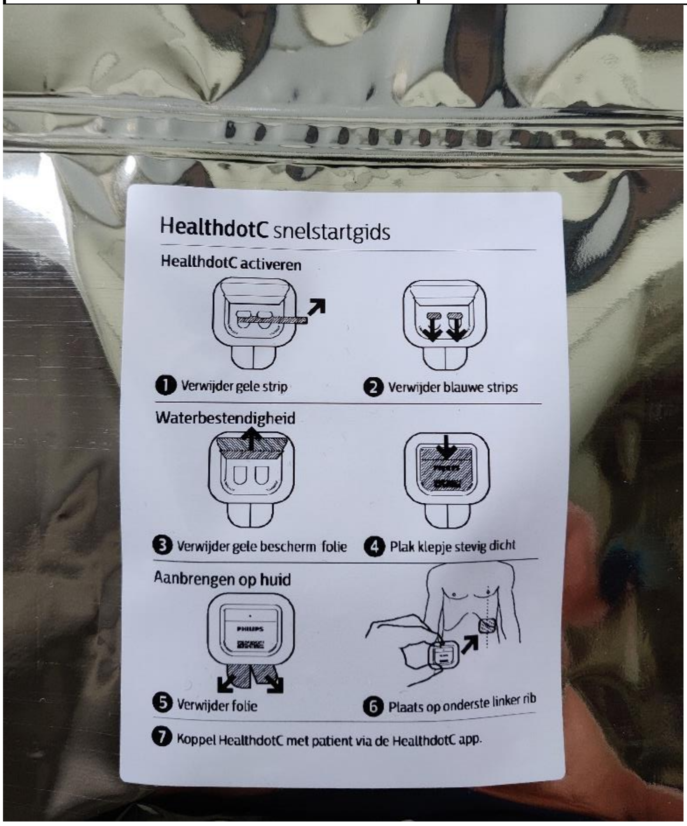

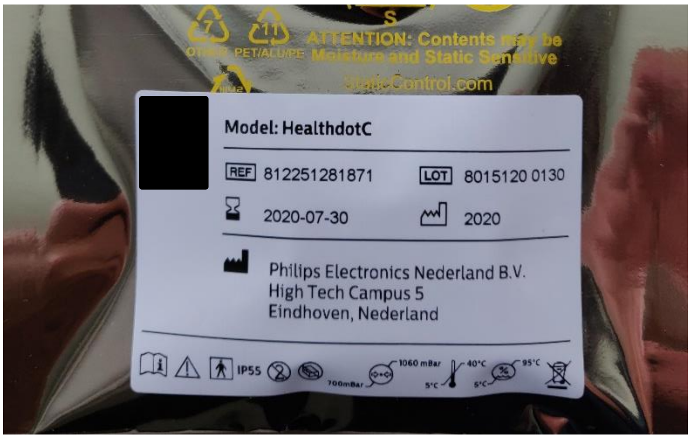

2.2 Intended purpose

The Healthdot wearable and backend is indicated for use by health care professionals in combination with a clinical information management system whenever there is a need for acquisition of the patient parameters respiratory rate and heart rate.

2.3 Device Description

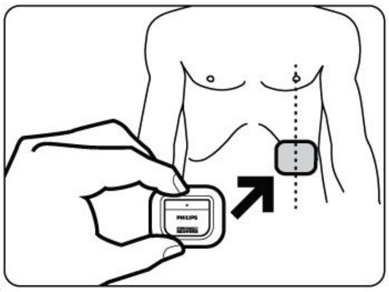

The Healthdot device (

Figure 1~~Figure 1~~) is a body worn (chest) accelerometer based measurement device, suitable for 24/7 data collection. The device is intended to be worn attached to the skin by means of a skin adhesive patch, typically placed on the left lower rib.

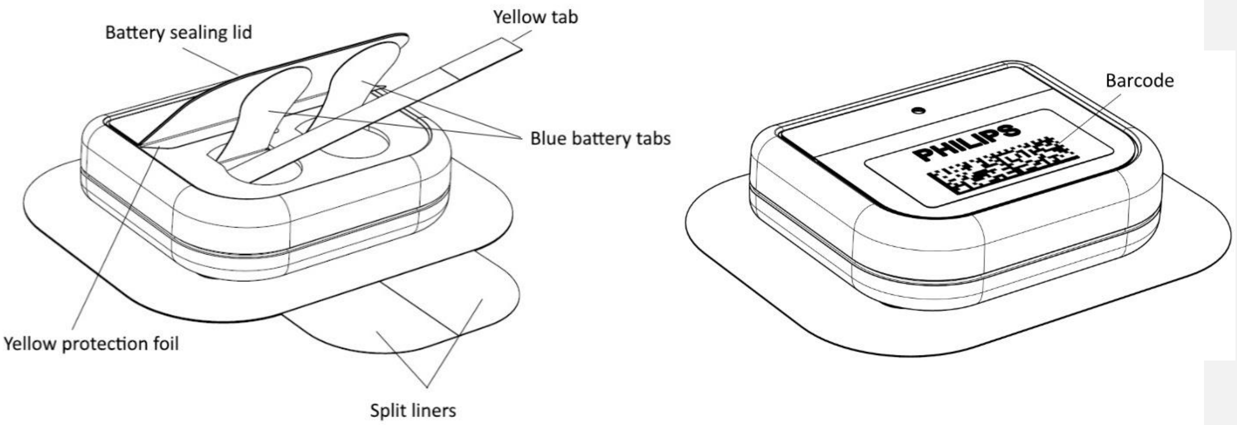

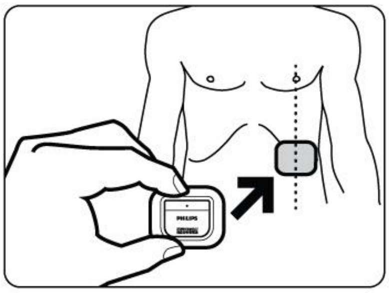

Figure 1: The Healthdot device and area where it is placed

A full device description can be found in the document D000385367 Healthdot Device Description. The general operating principle is based on continuous collection and periodic transmission of heart rate, respiratory rate, activity and posture by means of processing of accelerometer signals. These values are transferred from the Healthdot device via the KPN-LoRa network, which transfers the data to a backend and makes the data available for viewing on a (online) dashboard (Figure 2~~Figure 2~~), as part of the Philips Guardian system. The transmit interval is typically once every 5 to 15 mins.

Met opmaak

Met opmaak

Met opmaak

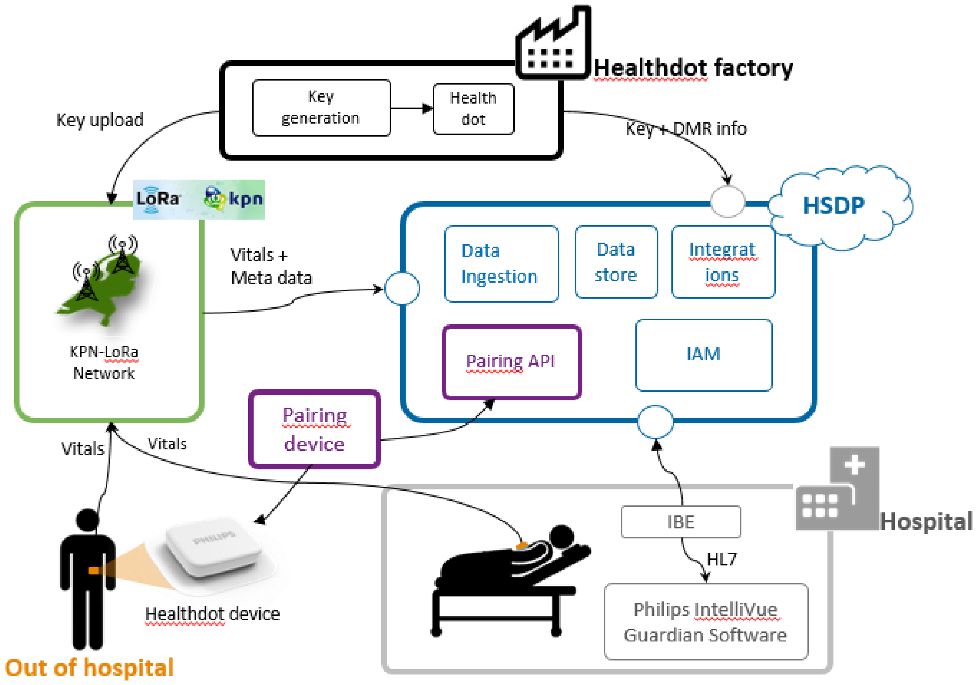

Figure 2: Healthdot system

3 JUSTIFICATION

The implementation of Enhanced Recovery After Bariatric Surgery (ERABS) has significantly reduced length of hospital stay (LOS) with no significant influence on overall morbidity or specific complication rates [1]. Currently discharge on postoperative day (POD) 1 is considered normal. Potential benefits of earlier discharge are improved patient and health professional satisfaction, and reduced healthcare costs. It has been shown in the literature that outpatient gastric sleeve [2] and gastric bypass [3] can be perfomed safely and with a low complication rate.

In a previous study (TRICA study; NCT03923127) which is currently executed and will be closed before this study here (PEACH) will start (Last patient last visit expected in August 2020), 350 patients have been included and worn the Healthdot in hospital after standard bariatric and large abdominal surgery. For 39 of these 350 patients overall, gold standard vital sign measurements of heart rate and respiratory rate have been recorded by electrocardiogram (ECG) and capnography respectively and compared with the Healthdot measurements to determine accuracy of the Healthdot system. An interim analysis shows that the Healthdot has an average error of 0.67 beats per minute (bpm) for heart rate on an aggregated level with 88.9% of all measurements below a deviation of 3 bpm to the gold standard ECG measurement. Additionally, the average error of breathing rates is -0.37 breath per minute (brpm) and 93.5% of all measurements below a deviation of 3brpm compared to capnography [4]. These data show that the Healthdot provides accurate measurements in view of its intended use. The here described PEACH study is the next step to validate the clinical usefulness of the Healthdot system by modifying the recovery path of bariatric patients after standard surgery by sending them home on the same day of surgery and comparing the outcome to the current recovery path, meaning the patients stay in hospital at least one night.

The most important reason to keep the patient in the hospital for one day is the detection of short-term complications such as bleeding. A remote monitoring system would allow the monitoring of vitals signs

in patients that have been discharged after standard surgery for outpatient recovery. This potentially facilitates detection of postoperative complications. The clinical investigation is designed to evaluate whether sending patients home on the same day of surgery can be safely done, combined with remote monitoring, and is not inferior to the current recovery path which means staying on night in hospital and leave the next day for home.

This clinical investigation is needed, to evaluate the possibility to send patients home one day early after bariatric surgery without adding additional risks to the patient. This study adds information on feasibility and outcome of outpatient recovery after standard bariatric surgery with Healthdot monitoring and this information is not available in clinical literature. The surgery itself and all activities performed during the stay in hospital (except for placing the Healthtdot) is standard of care and not part of the study procedures as such.

4 RISKS AND BENEFITS ASSESSMENT

☒ I have created and uploaded a risk management summary matrix

☒ I have created and uploaded a risk management plan

A summary overview of the risks and benefits of the investigational device and the clinical procedure applied in this clinical investigation are listed in the table below.

**Anticipated clinical benefits**	For the patients participating in this investigation no direct benefits have been identified. A potential benefit is that patients who are in the active group wearing the Healthdot are able to leave hospital one day early which could potentially lead to a higher patient satisfaction. Assessing patient satisfaction is one of the endpoints in this study.
**Anticipated adverse device effects**	The only anticipated adverse device effect is an allergic reaction to the plaster used to locate the device on the skin of the patient. This is mitigated by excluding patients with known allergies to these kind of medical adhesives.
**Residual risks associated with investigational device [as identified in risk analysis report]**	The residual risks associated with the devices and their potential interference with the clinical work flow have been identified, scored and mitigated. From the risk analysis it is concluded that the residual risks associated with the devices are acceptable.<br><br>The residual privacy risks associated with the study are that the devices with heart rate and respiratory rate data on it could come of and fall in the hands of unauthorized people.
**Risks associated with participation in clinical investigation**	Risks for participating in this investigation have been identified, assessed and mitigated. The residual risks are detailed in the Risk management Summary Matrix and are all deemed acceptable. Though the patients undergo surgery with the associated risks of this procedure this is not part of the study and the study does not influence the surgery risks. Sending the patient home on the same day has been assessed in bariatric surgery in several studies and risks associated with this are

	considered low. The placement of the devices will add some additional burden to the patient not related to her/his stay in hospital, however this additional burden is low.
**Possible interactions with concomitant medical treatments**	There are no interactions with concomitant medical treatments. If additional diagnostic procedures like CT, MRI are necessary the Healthdot will be removed before this procedure as also indicated in the instructions for use. If an X-ray is necessary, the Healthdot only needs to be removed if it is expected to compromise the X-ray image (e.g. X-ray is done at the location the Healthdot is attached).
**Steps that will be taken to control or mitigate risks**	**The mitigations implemented to adress study specific risks are detailed in the Risk management Summary Matrix which is part of this study dossier.**<br><br>**Mitigations related to skin issues are:**<br>1. Use of certified materials in contact with the skin.<br>2. Test of devices with healthy volunteers yielded highly favourable outcome on comfort and wearability.<br>3. Exclusion of patients with known allergies to skin adhesives.<br>4. Patient can take off the devices and stop participation.<br><br>**Mitigations related to privacy risk are:**<br>1. Data on the device will be encrypted.<br><br>The device will contain vital signs data but will not contain data that can be used to identify a patient (e.g.; patient name, address, etc.).
**Risk-to-benefit rationale**	After re-evaluation of the risks, the overall residual risk has been deemed acceptable. As an overall result we conclude that due to the low burden and the acceptable risks it is justified to conduct this study.

5 OBJECTIVES AND HYPOTHESES

This chapter describes the objectives and hypotheses of the clinical investigation, as well as the claims and intended performance of the investigational device to be verified, and the risks and anticipated adverse device effects to be assessed.

Primary objective:
To evaluate whether performing outpatient recovery after standard bariatric surgery is not inferior to the current recovery path based on a combined outcome measure.

Secondary objective(s)

- To assess Patient satisfaction (on a scale of 1-10) in both groups
- To assess feasibility of outpatient recovery after standard bariatric surgery by evaluating recruitment rate, adherence to protocol and randomization, and the amount of missing data.

- To evaluate the percentage and total number of false-positive notifications from the Healthdot system.
- To evaluate the percentage of missed events (i.e. false negatives) from the Healthdot system.
- To evaluate the total number of missed minor events from the Healthdot system.
- To evaluate the outcome of patients who choose HD versus patients who were randomized to HD on a combined outcome measure as defined for the primary endpoint.
- To compare the number of adverse events in both groups
- To compare the use of pain medication during day of surgery until evening in both groups.
- To compare the clinical decisions made on basis of remote monitoring with the Healthdot system, to decisions which also include information from a telephone consultation
- To evaluate the impact of outpatient recovery after standard bariatric surgery and the Healthdot on patient and health professional satisfaction
- To evaluate the costs involved with outpatient recovery after standard bariatric surgery supported by Healthdot.
- To evaluate usability of the visualization of Healthdot data on the Guardian dashboard.

Primary hypotheses
Outpatient recovery after standard bariatric surgery is not inferior to the current treatment based on a combined outcome measure.
The outcome measure that we will use for the analysis is a combined measure, based on the following:

- Mortality
- Severe postoperative complications (Clavien-Dindo IIIb or higher)
- Readmission (at least one night in hospital)
- Mild complications (Clavien-Dindo II and IIIa)
- Prolonged length of stay (3 days or more in hospital)

within 30 days after primary surgery.

This hypothesis will be accepted or rejected based on the outcome of the primary endpoint of this clinical investigation (for more details see page 33, Statistical considerations, Pass/fail criteria).

Secondary hypotheses
- Patient satisfaction >6 points (on a scale of 1-10) is not significantly different between both groups
- Outpatient recovery after standard bariatric surgery with Healtdot monitoring is feasible based on evaluating recruitment rate (>60% of patients), adherence to protocol (>75% of patients), and the amount of missing data (<10%).
- False-positive notifications from the Healthdot system are lower than 10% of notifications per patient.
- Missed events (false negatives) i.e. there was no notification from the Healthdot system when the patient needed to go to the hospital, are lower than 5% of all notifications.
- Missed minor events occur less than five times per patient. Missed minor events are defined as when there was no notification from the Healthdot system and the patient contacted the hospital, but there was no need for the patient to visit the hospital.
- The outcome of patients who choose HD versus patients who were randomized to HD on a combined outcome measure as defiend for the primary endpoint are the same.
- The number of adverse events is not significantly different in both groups.

- The amount of pain medication during day of surgery until evening in both groups is not significantly different.
- Clinical decisions made on basis of remote monitoring with the Healthdot system do not change in more than 5% of cases when information from a telephone consultation is also included in the decision making process.
- Patient and health professional satisfaction (on a scale from 1-10) for outpatient recovery after standard bariatric surgery with the Healthdot are equal or higher than for current treatment.
- The costs involved with outpatient recovery after standard bariatric surgery supported by Healthdot is less than the cost involved with the current recovery path, in the period up to 7 days after surgery.
- The usability of the visualization of Healthdot data on the Guardian dashboard is scored good (at least 4 out of 5).

These hypotheses will be accepted or rejected based on the outcome of this clinical investigation.

Claims and intended performance
In this investigation it will be tested if the Healthdot is suitable as a tool for out of hospital monitoring enabling a change in standard of care workflow for patients after surgery. Depending on the outcome claims may be derived from the primary and secondary hypothesis.

6 CLINICAL INVESTIGATION DESIGN

6. 1 General

This clinical investigation is designed as a single center patient preference trial in a tertiary hospital in the Netherlands. We will test for non-interiority of the outcome of the outpatient recovery after standard bariatric surgery compared to the current treatment, as well as evaluate the feasibility of the outpatient recovery after standard bariatric surgery.

To avoid bias, patient preference for either the current recovery path (group B) or the outpatient recovery (group A) after standard bariatric surgery is noted to be able to include the preference factor in the analysis. The patients that don't have a preference will be randomly assigned. The number of patients included in each group will be equal.

The primary endpoint of the study is the combined outcome measure, based on the following:

- Mortality
- Severe postoperative complications (Clavien-Dindo IIIb or higher)
- Readmission (at least one night in hospital)
- Mild complications (Clavien-Dindo II and IIIa)
- Prolonged length of stay (3 days or more in hospital)

within 30 days after primary surgery.

The secondary endpoints of the study are:

- Patient satisfaction >6 points (on a scale of 1-10)
- Percentage of patients recruited for the outpatient recovery after standard bariatric surgery group
- Percentage of patients with full adherence to protocol and randomization
- Percentage of missing data

- Percentage of false positive of the positive notifications from the Healthdot system
- Total number of false positive of the positive notifications from the Healthdot system
- Total number of false negative notifications from the Healthdot system
- The outcome of patients who choose HD versus patients who were randomized to HD on a combined outcome measure as defiend for the primary endpoint.
- To evaluate the outcome of patients who choose HD versus patients who were randomized to HD on a combined outcome measure as defiend for the primary endpoint.
- Number of adverse events in both groups
- Amount of pain medication taken on the day of surgery in both groups
- Percentage of clinical decisions made based on the Healthdot system, that changed when information from a telephone consultation was also included.
- Patient and health professional satisfaction (on a scale from 1-10) for both the outpatient recovery after standard bariatric surgery group as the current recovery path group
- The costs involved with outpatient recovery after standard bariatric surgery supported by Healthdot and the cost involved with the current recovery path, in the period up to 7 days after surgery.

For each patient, the parameters mortality, severe postoperative complications (Clavien-Dindo IIIb or higher), readmission (at least one night in hospital), mild complications (Clavien-Dindo II and IIIa) and prolonged length of stay (3 days or more in hospital) within 30 days after primary surgery will be determined from the patient medical record by the principal investigator. Patient satisfaction will be obtained through a questionnaire at the end of the 8-day study period. Healthcare professional satisfaction will be obtained through questionnaires after all patients have completed the study. Percentage of missing data will be determined at the end of the study. False positive and false negative notifications from the Healthdot system will be noted by the healthcare professional that receives the notifications. Clinical decisions made based on the Healthdot system, that changed when information from a telephone consultation was also included, will be noted by the healthcare professional that makes the decisions. The costs will be estimated for each patient, based on the events that occurred as noted in the patient medical file. The principal investigator will retrieve this information from the patient medical file. Costs calculation will include hospital stay, additional contacts, re-admission-hospital stay for mild complications (less than Clavien Dindo 3, like superficial woundinfection, dehydration, pain or worriness patient).

6.2 Investigational device(s) and comparator(s)

The Healthdot system consists of a wearable data logger that transmits data to be visualized on a dashboard. The data logger device consists of an adhesive layer, electronics and a battery. It contains an accelerometer which measures movement of the chest including movement of the lung and heart, processes these movement data with a validated algorithm to calculate heart rate, respiratory rate, posture and activity which are stored on the device and send out through the KPN-LoRa network to a backend at Philips. These data are then collected via an interface by the Philips Intellivue Guradian clinical information management system and are displayed via this dashboard inside the hospital. No comparator devices are used in this study.

6.3 Subjects

Inclusion criteria for subject selection

- Adult (equal or above 18)
- Approval for primary bariatric surgery (gastric sleeve or bypass) by a multidisciplinary bariatric team

- Willing and able to sign informed consent form
- Able to understand instructions
- In possession of a telephone on which patient can be reached for the duration of participation (day 1-8)
- An adult person must be present at the same location as the patient during the first night following surgery who is able to mobilize help or seek medical care if necessary.

Exclusion criteria for subject selection

- Patients of psychiatric wards, inmates of prisons, or other state institutions
- Investigator or any other team member involved directly or indirectly in the conduct of the clinical study
- Any skin condition, for example prior rash, discoloration, scars or open wounds at the area (Left lower rib) where the Healthdot needs to be placed
- Known allergy for the tissue adhesive used in the Healthdot (white band-aid)
- Use of topical that is known to influence the skin at the test area (such as medical and non-medical creams or lotions)
- Patient with active implantables such as Implantable Cardioverter Defibrilator (ICD) and pacemaker
- Expected participation less than 8 days
- Left lower rib (place where Healthdot will be applied) is involved in the area of surgery, area of disinfection or area where bandages are needed.
- Patients with antibiotic resitant infections (e.g. MRSA)

Enrollment schedule

The point of enrollment is the time at which, following recruitment, a subject signs and dates the informed consent form. The first subject is expected to be enrolled in September 2020. The Clinical Investigation is expected to take 8 months. The duration of the active participation of each subject is 8 days.

In this clinical investigation, 200 patients will be enrolled. The enrolment period will be 6-7 months. Half of the patients will be assigned to the outpatient group, and half of the patients will be assigned to the regular treatment group. Patients included in the study can choose in which group they want to be. This way they can choose whether they receive outpatient recovery or the regular treatment. If the preferred group is full, the patient can choose to participate in the other group, or to withdraw from the study. If the patient has no preference, he/she is randomly assigned to either treatment, or if one group is full, to the treatment for which 100 patients have not yet been included. Patient preference is recorded.

Criteria and procedures for subject withdrawal or discontinuation

Subjects can leave the study at any time for any reason if they wish to do so without any consequences. The investigator can decide to withdraw a subject from the study for medical reasons.

If one of the following criteria is fulfilled, the participation of a subject to the study is terminated:

- Withdrawal of informed consent
- Serious adverse events that were judged by the investigator to have a reasonable possibility
- that the event may have been caused by the investigational device
- Investigator’s decision for any safety reasons and/or medical judgment in the best interest of patients' health

These subjects need to be replaced if dropout occurs before the second post-surgical day.

6.4 Procedures

Specimen Banking (saving human fluid or tissue for future research)

☒ Not applicable—this is a human study without specimen banking.

Clinical procedures

Patient screening/inclusion

The patients are recruited during the pre-operative consultation at the obesity centre. Patients are informed about the study and will be asked if they are interested in participating in a study in which they could either receive regular treatment (stay at least one night in hospital) or undergo standard f care surgery and leave the hospital on the same day while being monitored with use of the Healthdot. Interested patients are handed out the information letter including the informed consent form. If the patient agrees to participate and signs informed consent he/she can be enrolled into the study. The patient can choose whether he/she wants to be assigned to outpatient recovery or the regular recovery path. If the preferred group is full, the patient can choose to participate in the other group, or to withdraw from the study. If the patient has no preference, he/she is randomly assigned to either recovery path. Randomization is done by computer software. Howerver, if one group is already full, the patient will be assigned to the group for which 100 patients have not yet been included. Patient preference is recorded. As demographics the following data of the patient are collected: Age, height, weight, gender.

Start of the study

The activities in both groups are outlined in

Table 1

~~Table 1~~, with the differences between the two groups described for clarity. Patients who have chosen to undergo outpatient recovery will be scheduled for surgery on the earliest available time-slots (before 12:00). The Healthdot will be applied mid-clavicular on the lowest left rib of the chest after surgery. HealthdotThe Healthdot can be removed by the patient themselves in case of emergency with a remover tissue which will be handed out to the patient before discharge. The patient will be instructed on how to remove the Healthdot with the tissue. The postoperative standard care will be arranged in such a way that same day discharge is possible for this group of patients.
Patients who have chosen the regular recovery path will receive care in accordance with current protocol.

Met opmaak
Met opmaak
Tekstkleur: Au
Met opmaak
Met opmaak

Discharge from hospital

Patients in the outpatient recovery group will be released from hospital on the discretion of a nurse under supervision of a physician and return to their homes wearing the Healthdot. The Healthdot calculates and transmitts heart rate, respiratory rate, posture and activity level. The physician will be able to view the values for these four parameters through a dashboard. On the dashboard, events with a heart rate above a pre-defined threshold will be flagged. A teleconsultation will be scheduled for the following morning (day 2). Before the teleconsultation, the Healthdot data will be interpreted by the physician who will then assess whether additional action is indicated. The decision based on Healthdot data is noted in the eCRF. After the teleconsultation, it is noted whether additional or

different decisions are made about the treatment based on the additional information from the teleconsultation.
On day 3-8 the physician will check the Healthdot dashboard daily. The physician may decide to have a telephone consultation, e.g. when the Healthdot dashboard flags an event. If the physician will determine that additional action is needed (e.g. based on the information on the dashboard, or when the patient calls), in the absence of an indication from the Healthdot, such an event will be viewed as a false negative. It will be retrospectively assessed, based on the patient medical record, if false negative events have occurred. For all events flagged in the Healthdot dashboard, the physician will record whether it was a false positive notification or a true positive notification in the eCRF.
Patients in the regular recovery group will be released from hospital on the discretion of a nurse under supervision of the physician the day after surgery. For these patients no appointments outside of the regular protocol are planned.

Table 1: Overview of protocol activities in the hospital for the two patient groups, and the differences

Protocol Activity for patient group "Outpatient recovery"	Protocol Activity for patient group "Regular recovery"	Differences
**Before start of the study**		
- Inform patient about study<br>- Signature of informed consent form<br>- Verification of inclusion and exclusion criteria	- Inform patient about study<br>- Signature of informed consent form<br>- Verification of inclusion and exclusion criteria	No difference.
**Day 1**		
Surgery, between 8:00h and 12:00h	Surgery	There is no difference in surgery since this is not part of the study. Surgery for patients leaving the same day is only scheduled in the morning.
By nurse, in the recovery room:<br>- Application of the Healthdot device, if not already placed 5 days before surgery		Patients leaving hospital the same day receive the Healthdot device.
By nurse, upon arrival to the ward:<br>- Anamnesis<br>- In first 3 hours, an hourly check of blood pressure and hart rate<br>- Remove intravenous drip after 2L<br>- Administer fragmin injection (blood thinner)<br>- Start mobilising	By nurse, upon arrival to the ward:<br>- Anamnesis<br>- In first 3 hours, an hourly check of blood pressure and hart rate<br>- Remove intravenous drip after 2L<br>- Administer fragmin injection (blood thinner)<br>- Start mobilising	No difference, standard of care.

- Start with sips of water, expanding to clear liquids	- Start with sips of water, expanding to clear liquids	
By nurse, between 16:00h and 17:00h:<br>- Check Eearly Warning Score (EWS) and pain score<br>- Inspecting the wound<br>- Ensure micturition within 6 hours after surgery<br>- Explain and instruct patient about fragmin (blood thinner) injections (to be used for 4 weeks)<br>- Explain postoperative lifestyle/nutritional advice/upcoming appointments		The activities that occur at this point in the patients leaving on the same day is part of the standard of care but performed earlier that day. This willalso happen later in the regular recovery group.
By nurse, under supervision of physician, around 20:00h:<br>- Anamnesis<br>- Physical examination<br>- Determine hemoglobin<br>This will be followed by discharge if criteria set by physician are met.	By nurse, around 20:00h:<br>- Check EWS and pain score<br>- Inspecting the wound<br>- Ensure micturition within 6 hours after surgery<br>The patient will be observed during the night by the nurse.	The outpatient group is evaluated for discharge. This will happen on day 2 for the regular treatment group. The procedures for both groups are the same and standard of care.
**Day 2**		
By physician, around 9:30h:<br>- Open the Healthdot dashboard to check vitals and see if there are notifications<br>- Perform telephone consultation<br>- Make decision about care pathways, i.e. if action is needed	By nurse, between 7:00h - 8:00h<br>- Anamnesis<br>- Check EWS and pain score<br>- Inspecting the wound<br>- Encourage fluid intake (at least 500ml before 12:00h)<br>- Explain and instruct patient about fragmin (blood thinner) injections (to be used for 4 weeks)<br>- Explain postoperative lifestyle/nutritional advice/upcoming appointments	The patients from the outpatient recovery group are at home and will be remotely monitored and receive a telephone consultation. The patients from the regular recovery group are in the hospital and are monitored there by the nurse.
	By nurse, supervised by medical resident, around 8:30h:<br>- Anamnesis<br>- Physical examination	The regular recovery group is evaluated for discharge at this point.

	- Determine hemoglobin	
	Nurse, around 12:00h:<br>- Anamnesis<br>- EWS<br>- Check if fluid intake has been at least 500ml<br>- Explain postoperative lifestyle/nutritional advice/upcoming appointments<br>This will be followed by discharge if criteria set by physician are met.	The explanation on lifestyle etc. is given on day 1 for the outpatient recovery group.<br>The evaluation for discharge happened on day 1 for the outpatient recovery group.
**Day 3-8**		
Patient calls hospital in case of pain or concern.<br>Physician checks the Healthdot dashboard for notifications daily, and can decide to take actions based on this information.	Patient calls hospital in case of pain or concern.	The outpatient recovery group is monitored remotely with the Healthdot system.

Dashboard usability

When the caregivers have been using the dashboard for at least two months, an interview session with the users will be held. This will take approximately one hour, and will be performed at the location where the dashboard is typically used. Alternatively, if this location is not available (e.g. Covid-19 measures), the interview session will be held at another location (or online) where the dashboard can be accessed online. It will be a semi-structured interview, to asses at least the following points:

- How did they experience working with this interface
- What works well
- How could the interface be improved
- Do they miss functionality
- How much time do they need per virtual round
- How much time do they need to call a patient

The interview will be done in person (if possible due to Covid situation) and documented by the resrearcher on paper.

Follow up

Patients who received the Healthdot will remove this themselves on day 8. They use a return envelope, which was given to them at discharge, to send back the device to Philips.

Patients in both groups will be requested to fill in a short satisfaction questionnaire on day 8. This questionnaire was given to them at discharge and should be sent to Philips together with the Healthdot device or a provided envelope without the device for the control group. Health care professionals will be requested to fill in a similar satisfaction questionnaire after the entire study is

completed. The presence or absence of readmission within 30 days will be observed from the patient record.

The questionnaires for the patient and the health care professional can be found in Table 2~~Table 2~~ and Table 3~~Table 3~~, respectively.

Met opmaak

Met opmaak

Table 2: Patient satisfaction questionnaire. Patient should circle the selected answer.

<table>
<tr><td>How satisfied are you with the frequency of medical interactions?</td><td colspan="2">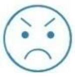</td><td colspan="2">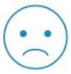</td><td colspan="2">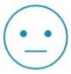</td><td colspan="2">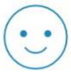</td><td colspan="2">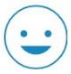</td></tr>
<tr><td colspan="11"></td></tr>
<tr><td>If you were not satisfied, is the frequency of medical interactions either too low or too high?</td><td colspan="5">Too low</td><td colspan="5">Too high</td></tr>
<tr><td colspan="11"></td></tr>
<tr><td>How satisfied are you with the length of time you stayed in the hospital?</td><td colspan="2">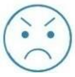</td><td colspan="2">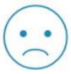</td><td colspan="2">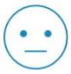</td><td colspan="2">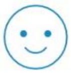</td><td colspan="2">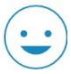</td></tr>
<tr><td colspan="11"></td></tr>
<tr><td>If you were not satisfied, was the length of time you stayed in the hospital either too long or too short?</td><td colspan="5">Too short</td><td colspan="5">Too long</td></tr>
<tr><td colspan="11"></td></tr>
<tr><td>How satisfied are you with the level of care after discharge from the hospital?</td><td colspan="2">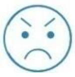</td><td colspan="2">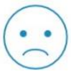</td><td colspan="2">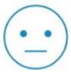</td><td colspan="2">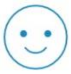</td><td colspan="2">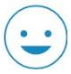</td></tr>
<tr><td colspan="11"></td></tr>
<tr><td>What is your overall level of satisfaction?
(1 “not satisfied at all” to 10 “very satisfied”)</td><td>1</td><td>2</td><td>3</td><td>4</td><td>5</td><td>6</td><td>7</td><td>8</td><td>9</td><td>10</td></tr>
<tr><td colspan="11"></td></tr>
<tr><td>Did you find it important that you had the possibility to choose between going home sooner with a sensor or staying a day longer in the hospital without a sensor?</td><td colspan="3">No</td><td colspan="4">Yes</td><td colspan="3">Don’t know</td></tr>
<tr><td colspan="11"></td></tr>
<tr><td>Do you have any comments about how you experienced the home monitoring? Do you have any suggestions for improvement? (Only fill in this question if you did wear the investigational device)</td><td colspan="10"></td></tr>
</table>

Table 3: Health care professional questionnaire. Health care professional should circle the selected answer.

How satisfied are you with the frequency of interactions with the regular treatment patient group?	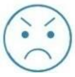		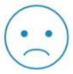		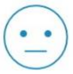		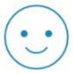		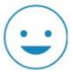	
If you were not satisfied, was the frequency of interactions with the regular treatment patient group either too low or too high?	Too low					Too high				
How satisfied are you with the amount of time you spent on the regular treatment patient group?	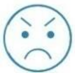		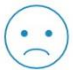		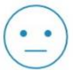		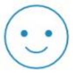		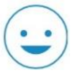	
If you were not satisfied, was the amount of time you spent on the regular treatment patient group either too long or too short?	Too short					too long				
How satisfied are you with the method of care after patient discharge in the regular treatment patient group?	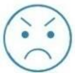		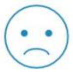		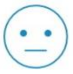		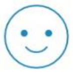		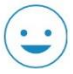	
What is your overall level of satisfaction of the care pathway for the regular treatment patient group?	1	2	3	4	5	6	7	8	9	10
How satisfied are you with the frequency of interactions with the outpatient recovery group?	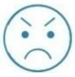		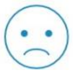		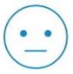		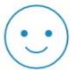		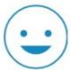	
If you were not satisfied, was the frequency of interactions with the outpatient recovery group either too low or too high?	Too low					Too high				
How satisfied are you with the amount of time you spent on the outpatient recovery group?	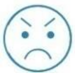		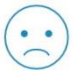		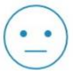		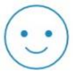		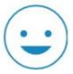	
If you were not satisfied, was the amount of time you spent on the outpatient recovery group either too long or too short?	Too short					too long				
How satisfied are you with the method of care after patient discharge in the outpatient recovery group?	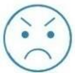		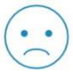		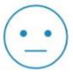		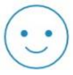		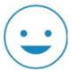	

How satisfied are you with the Guardian dashboard?	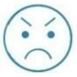		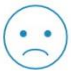		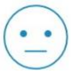		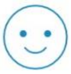		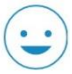	
What is your overall level of satisfaction of the care pathway for the outpatient recovery group?	1	2	3	4	5	6	7	8	9	10

Schedule of activities

The Schedule of Activities table provides an overview of the protocol visits and procedures. Refer to Clinical Procedures for detailed information on each procedure and assessment required for compliance with the protocol.

Schedule of Activities

**Protocol Activity**	D0 (can be up to 2 weeks before D1)	Interim Visit (at least 5 days before surgery appointment)	D1	D2	D3-8	D8	D31	After 2 months	After all patients
Signature of informed consent form	X								
Verification of inclusion and exclusion criteria	X								
Demographics	X								
Outpatient recovery protocol in hospital			X						
Regular recovery protocol in hospital			X	X					
Teleconsultation for outpatient recovery group				X					
Telemonitoring for outpatient recovery group				X	X				

Patient calls to hospital in case of pain or concern				X	X				
Patient removes Healthdot. Patient fills in questionnaire.						X			
End of study						X			
Interview about dashboard with Healthcare professionals								X	
Healthcare professionals fill in questionnaire									X
CRF completion, including 30-day readmission							X		
Adverse event documentation	X		X	X	X	X			
Device accountability logging			X			X			

Sponsor activities
The sponsor will provide the Healthdot system, and instruct the investigators on how to handle the Healthdot device, where to position it, and how to use the system. The Sponsor will provide the infrastructure to view the transmitted signals on the dashboard inside the hospital.

6.5 Monitoring plan

☐ Study does not require on-site monitoring. Justify here (e.g., non WMO study in NL); consult with Q&R Manager as needed:

The monitoring plan to be followed, including access to source data and the extent of source data verification planned, is described in a separate document.

7 STATISTICAL CONSIDERATIONS

Study population
All patients who are randomized will be included into the analysis and analyzed according to the group they were originally assigned to, regardless of what treatment (if any) they received (intention to treat analysis).

Statistical design

This study is primarily designed to test for non-inferiority of outpatient recovery compared to regular treatment for bariatric surgery. The outcome measure that we will use for the analysis is a combined measure, based on the following:

- Mortality
- Severe postoperative complications (Clavien-Dindo IIIb or higher)
- Readmission (at least one night in hospital)
- Mild complications (Clavien-Dindo II and IIIa)
- Prolonged length of stay (3 days or more in hospital)

within 30 days after primary surgery.

We choose to use this combined outcome measure since it is in line with other surgical studies [5] making it comparable to literature.

I current practice at Catharina Hospital, Eindhoven, 95% of all bariatric surgery patients do not have any of these events which is considered the textbook outcome here.

We set the following null and alternative hypotheses for proving non-inferiority:

H0: $p_{regular} - p_{outpatient} \geq \Delta$
H1: $p_{regular} - p_{outpatient} < \Delta$

Where $p_{regular}$ is the textbook outcome proportion in the regular treatment group, and $p_{outpatient}$ is the textbook outcome proportion in the outpatient recovery group. We will use a non-inferiority margin (Δ) of 7%, which is deemed clinically significant by the Principal Investigator. This is also justified by a previous study where the average textbook outcome across bariatric surgery patients in the Netherlands was 88,7% [5] which helps to derive a non-inferiority margin of around 7% to the one seen at Catharina Hospital. We will use an uncorrected chi-squared statistic. Based on the sample size calculation done below an p value of 0.05 will be considered statistically significant. For the interim analysis an alpha correction for multiple testings is applied and a p value of 0.025 will be considered statistically significant.

The secondary endpoints will be analysed statistically in analogy to the primary endpoint by uncorrected chi-squared statistic where deemed appropriate.

Sample size

Sample size calculations were performed using PS: Power and Sample Size Calculation software. With a power of 0.80, an α of 0.05, a non-inferiority margin of 0.07 and an expected proportion of 0.96, this resulted in a sample size of 97 (we choose 100) patients per group when using an uncorrected chi-squared statistic.

Missing Data

No replacement of missing data will be performed and the affected measurements will be regarded as lost for final statistical analysis.

Demographic Data

Demographic variables (e.g. age, gender, BMI) will be given for each defined analysis population. Data will be summarized using frequency distributions (number and percentage) for categorical/ordinal variables and mean, standard deviation and range for continuous variables.

Expected drop-out rates
The expected drop-out rate is 10% (20 patients in this sample). Patients dropping out during the first 2 days of the study will be replaced until the final 200 patients are included and have participated more than the 2 days defined for replacement.

Pass/fail criteria
The study is considered successful if the primary endpoint is addressed and the non-inferiority of outpatient recovery compared to regular treatment for bariatric surgery can be concluded.

Interim analysis
The interim analysis will take place when data from 50 patients in both groups has been collected. Interim analysis will involve a comparison of the textbook outcome, as described above for the primary endpoint, between the two groups. The individual parameters of the textbook outcome will also be compared, i.e. discharge as planned, readmission in the 30 days after surgery, additional consultation by phone in the 7 days after surgery, additional consultation in the 7 days after surgery, and patient satisfaction. Based on the outcome of this interim analysis the investigators together with the sponsor will decide if the study needs to be stopped.

Termination of entire study on statistical grounds
Based on the interim analysis, the study could be stopped in case of unacceptable inferiority or unanticipated accumulation of adverse events increasing patient risks to an unacceptable level.

8 DATA MANAGEMENT

Electronic Case Report Forms

An eCRF will be used in this study and will be completed for each included subject. The eCRF used for this study is Research Manager, a standard tool used by the clinical site for all clinical investigations.

The investigator has ultimate responsibility for the collection and reporting of all clinical, and safety data entered on the eCRFs and any other data collection forms (source documents) and ensuring that they are accurate, authentic/original, attributable, complete, consistent, legible, timely (contemporaneous), enduring and available when required. The eCRFs must be (electronically) signed by the Principal Investigator or the assigned subinvestigator to attest that the data contained on the eCRFs is true. Any corrections to entries made in the eCRFs, source documents must be dated, initialed and explained (if necessary) and should not obscure the original entry. In case of electronic data record, the corrections history will be maintained by an audit trail.

In most cases, the source documents are the hospital's or the physician's subject chart. In these cases data collected on the eCRFs must match the data in those charts.

In some cases, the eCRF, or part of the eCRF, may also serve as source documents. In these cases, a document should be available at the investigator's site as well as at Philips and clearly identify those data that will be recorded in the eCRF, and for which the eCRF will stand as the source document.

Procedures

The code list, signed informed consent forms and contact information will stay at the hospital. Coded data will be transferred to a Philips project share.

Data retention
Personal data that will stay at the hospital will have a retention time of 15 years. Data stored on the Philips project share will be reviewed at least every 5 years for further retention.

9 AMENDMENTS

Any changes to the clinical study protocol after initial ICBE approval are documented as protocol amendments. After ICBE approval, and prior to execution at the study site, the amendment will be:

- submitted and approved by the applicable IRB/REC and regulatory authorities
- signed by the Investigator(s)

10 DEVIATIONS

Protocol deviations are any alteration or deviation from the ICBE-approved research plan as defined in the study protocol. This includes equipment failures during study procedures. The researcher will deviate from the protocol whenever necessary to protect the participant's health, rights or welfare and these types of deviations will be reported to ICBE and the external METC as soon as possible. Major deviations must be reported to ICBE and the external METC as soon as possible. In non-urgent/emergent situations, the researcher will obtain ICBE-approval for the planned deviation in advance of performing the changed activity.

If the researcher anticipates that there will be future requests for the same deviation, then the protocol will be amended (and such amendments must be approved by ICBE and the METC). A minor deviation is something that does not cause harm or have the potential to cause harm to the participant, and does not impact the integrity of the study. These shall be documented in the study file and uploaded to ICBE for their awareness. Other reasons for amendment requests include adding a study site, increasing the number of research participants, and extending the time to perform the study. If the study objectives and procedure or cohort changes or the study changes from non-medical to medical, this generates a new study (not an amendment).

Corrective and preventive actions and principal disqualification criteria
All protocol deviations will be documented and assessed. If required, corrective and preventive action will be agreed upon and implemented with the relevant site. These decisions, activities and possible preventive actions will be documented.

A Principal Investigator can be disqualified if he/she has repeatedly or deliberately failed to comply with the requirements as specified in the protocol, including compliance with the relevant regulations, or if he/she has submitted false information in any required report.

Corrective actions may include supplemental protocol training, discussions with PI and study staff for activities to prevent future recurrence, etc.. Misconduct can cause PI disqualification.

11 DEVICE ACCOUNTABILITY

The procedures for the accountability of investigational devices are in accordance with the Guidance.

The access to investigational devices will be controlled and the investigational devices will only be used in the clinical investigation and according to the CIP.

The sponsor shall keep records to document the physical location of all investigational devices from shipment of investigational devices to the investigation sites until return or disposal. This record will be based on device accountability form.

The principal investigator or an authorized designee shall keep records documenting the receipt, use, return and disposal of the investigational devices. This record shall include:

- the date of receipt,
- identification of each investigational device (batch number/serial number or unique code),
- the expiry date, if applicable,
- the date or dates of use,
- subject identification,
- the date on which the investigational device was returned/explanted from subject, if applicable, and
- the date of return of unused, expired or malfunctioning investigational devices, if applicable.

Devices and/or questionnaires will be returned by the patient in a provided envelope to the hospital. The devices will be wiped down with standard alcohol wipes and will be quranteened together with the questionnaire for 4 days at the hospital before return to Philips (Covid-19 measure).

12 STATEMENTS OF COMPLIANCE

The clinical investigation will be conducted according to the principles of the Declaration of Helsinki and other guidelines, regulations and Acts. The study will be conducted in accordance with the Medical Research Involving Human Subjects Act (WMO) and the ISO 14155 standard on Clinical investigation of medical devices for human subjects — Good clinical practice.

The clinical investigation shall not begin until the required approval/favorable opinion from ICBE and the METC and regulatory authority have been obtained. Any additional requirements imposed by ICBE and the METC or regulatory authority shall be followed.

Compensation for injury
Insurance for subjects participating in medical research that falls within the scope of the *WMO* is available in accordance with the legal requirements of article 7 of the *WMO* and *Medical Research (Human Subjects) Compulsory Insurance Decree* of 23 June 2003.
The sponsor/investigator has a liability insurance in accordance with article 7, subsection 6 of the *WMO*.

The sponsor (also) has an insurance in accordance with the legal requirements in the Netherlands (Article 7 *WMO* and the *Measure Regarding Compulsory Insurance for Clinical Research in Humans* of 23th June 2003). This insurance provides cover for damage to research subjects through injury or death caused by the study.

- € 650.000,-- (i.e. six hundred and fifty thousand Euro) for death or injury for each subject who participates in the Research;

- € 5000.000,-- (i.e. five million Euro) for death or injury for all subjects who participate in the Research;
- € 7.500.000,-- (i.e. seven million and five hundred thousend Euro) for the total damage incurred by the organization for all damage disclosed by scientific research for the Sponsor as ‘verrichter’ in the meaning of said Act in each year of insurance coverage.

The insurance applies to the damage that becomes apparent during the study or within 4 years after the end of the study.

13 INFORMED CONSENT PROCESS

☐ The requirement for informed consent has been waived (i.e., not required by law OR waived by IRB/REC/METC). Upload waiver if available.

☒ Written informed consent will be obtained from adult participants

☐ Assent will be obtained from children/minors

☐ Parental permission will be obtained when children are participating

☐ Surrogate consent will be obtained when participants lack decisional capacity

☐ Deferred consent will be obtained [e.g., emergency research]

☐ Click-through consent will be obtained [Internet/app-based research]

☐ Implied consent [i.e., consent through action such as taking a test]

13.1 Consent Process

Study participation is voluntary. Potential subjects, are given the most current ICBE/IRB/METC-approved consent form to read. They will be provided ample time (at least 2 weeks) for review and an opportunity to ask questions about the study.
If they agree to participate, they will sign the consent form and be given a copy of the signed document for their records. Each of these actions/steps will be documented. Only after Informed Consent has been obtained, may the remaining study procedures begin.

Eligibility process
After patients have signed up at the Obesity center, they undergo a screening to check if they are eligible for bariatric surgery. During this screening the nurse will also check inclusion criteria for the PEACH trial and, if eligible, will make a note about this in the electronic patient record. After screening, patients have a ‘commitment’ appointment. At this time patients will individually receive information about the study from a researcher who is not directly involved in the surgery or direct care of the patient. At least 2 weeks after this, if patients have been approved for surgery, they will have an appointment with the researcher who will check inclusion criteria and sign informed consent.

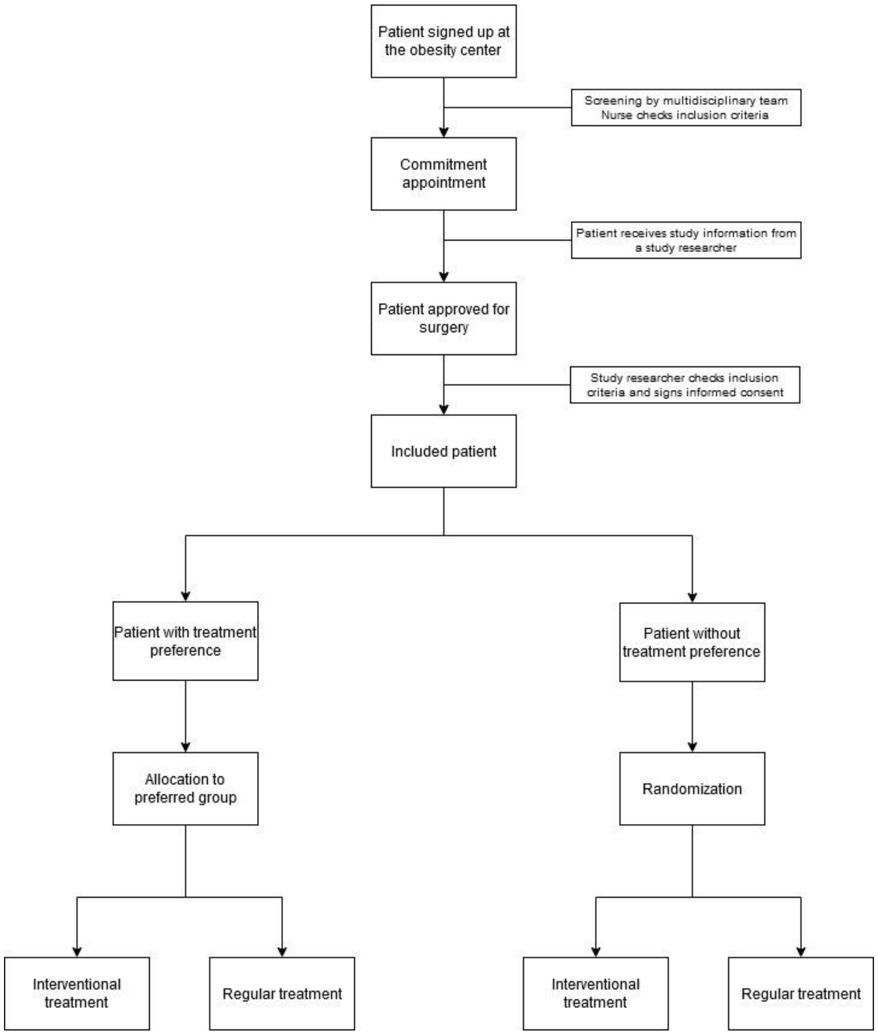

13.2 New Information about the Study

Any new information about the study that may affect a consented subject's decision to be in the study (e.g., changed procedures, safety, etc.), will be communicated in a timely manner. Depending on the nature of the new information, subjects who have completed the study may or may not be informed, documenting the decision and justification as well as any activities for informing completed subjects. Additionally, the approving IRB/METC and/or regulatory authority will also be informed.
The currently approved Consent Form will be updated and submitted to the ICBE and approving IRB/METC and/or regulatory authority for review and approval. Active subjects will be re-consented, following the above process, with the newly-approved consent form.

14 ADVERSE EVENTS AND DEVICE DEFICIENCIES

Definitions

Adverse events and adverse device effects, device deficiencies and serious adverse events and serious adverse effects and, unanticipated serious adverse device effects are defined in the table below:

Definition	***Description***
Adverse Event (AE)	Any untoward medical occurrence, unintended disease or injury, or untoward clinical signs (including abnormal laboratory findings) in subjects, users or other persons, whether or not related to the investigational medical device<br>NOTE 1 This definition includes events related to the investigational medical device or the comparator.<br>NOTE 2 This definition includes events related to the procedures involved.<br>NOTE 3 For users or other persons, this definition is restricted to events related to investigational medical devices.
Adverse Device Effect (ADE)	Adverse event related to the use of an investigational medical device<br>NOTE 1 This definition includes events related to the investigational medical device or the comparator.<br>NOTE 2 This definition includes events related to the procedures involved.<br>NOTE 3 For users or other persons, this definition is restricted to events related to investigational medical devices.
Serious Adverse Event (SAE)	Adverse event that<br>a) led to death,<br>b) led to serious deterioration in the health of the subject, that either resulted in<br>1) a life-threatening illness or injury, or<br>2) a permanent impairment of a body structure or a body function, or<br>3) in-patient or prolonged hospitalization, or<br>4) medical or surgical intervention to prevent life-threatening illness or injury or permanent impairment to a body structure or a body function,<br>c) led to foetal distress, foetal death or a congenital abnormality or birth defect<br><br>NOTE Planned hospitalization for a pre-existing condition, or a procedure required by the CIP, without serious deterioration in health, is not considered a serious adverse event.
Serious Adverse Device Effect (SADE)	Adverse device effect that has resulted in any of the consequences characteristic of a serious adverse event or that might have led to any of these consequences if suitable action had not been taken or intervention had not been made or if circumstances had been less
Unanticipated Adverse Device Effect (UADE)	Serious adverse device effect which by its nature, incidence, severity or outcome has not been identified in the current version of the risk analysis report<br>NOTE Anticipated serious adverse device effect (ASADE) is an effect which by its nature, incidence, severity or outcome has been identified in the risk analysis report.

Reportability

All adverse events shall be reported to the Study Manager. ADEs, SAEs, SADEs and USADEs shall be reported to the Q&R office as well (QandRoffice.pre@philips.com). The Director Q&R shall assess the ADEs, SAEs, SADEs and USADEs for reportability to Competent Authorities.

Gewijzigde v

Reporting process
Reporting shall include the date of the adverse event, research subject ID#, description of the adverse event, treatment, resolution, and assessment of both the seriousness and the relationship to the investigational device and study procedures.

Timelines
The time period in which the principal investigator shall report all adverse events and device deficiencies to the sponsor and, where appropriate, to ECs and the regulatory authority, and the details of the process for reporting device deficiencies, are detailed in the table below.

<table>
<tr><td rowspan="2">Category</td><td>Report to Study Manager</td><td>Report to Accredited Ethics Committee</td><td>Report to Q&R office
QandRoffice.pre@philips.com
Phone +31 40 27 95236 or +31 6 21459921
Fax +31 40 274 6321 (inform us after you send a fax)</td></tr>
<tr><td>by
Principal Investigator</td><td>by
Principal Investigator</td><td>by
Principal Investigator
by Study Manager</td></tr>
<tr><td>Adverse Event (AE)</td><td>Periodic reporting to Sponsor
Periodic collection by Sponsor</td><td colspan="2">As part of Sponsor’s Clinical Investigation Report</td></tr>
<tr><td>Adverse Device Effect (ADE)</td><td rowspan="4">Immediate, < 24 hr</td><td>As part of Ethics Committee Reporting timelines
or
< 24 hr following instructions by Sponsor</td><td rowspan="4">Immediate, <24 hr</td></tr>
<tr><td>Serious Adverse Event
(SAE)</td><td>As part of Ethics Committee Reporting timelines
or
< 24 hr following instructions by Sponsor</td></tr>
<tr><td>Serious Adverse Device Effect (SADE)</td><td>Immediate, < 48 hrs</td></tr>
<tr><td>Unanticipated Adverse Device Effect (UADE)</td><td>As part of Ethics Committee Reporting timelines
or
< 24 hr following instructions by Sponsor</td></tr>
</table>

Gewijzigde v

Foreseeable adverse events and anticipated adverse device effects
No anticipated adverse device effects have been identified. However, since the study described here aims at detecting deterioration of health in patients post surgery, the following adverse events not associated with this study but with surgery in general are expected:
- Bleeding
- Anemia
- Anastomotic leakage
- Infection: wound, abdominal, pneumonia, urinary tract
- Dehydration
- Delayed gastric emptying
- Thrombosis: cerebral or cardiac infarction, intestinal ischemia, lung embolism

According to the Inspectie voor de Gezondheidszorg (Aanmeldingsformulier klinisch onderzoek met medisch hulpmiddel, Bijlage E 2017-07-14) these adverse events even if considered serious are not reportable since they are expected and described here as long as they do not lead to the suspension or termination of the trial or result in a change of the investigational product.

Emergency contact details

		Joerg Liebmann<br>Philips Electronics B.V., Philips Research,<br>High Tech Campus 11<br>5656AE Eindhoven	
·	**E mail**:	**During Business hours (09:00-17:00):**<br>Joerg.liebmann@philips.com	
·	**Phone**:	**During Business hours** *(09:00-17:00)*:<br>+31-(0)628498405	**Outside Business hours:**<br>+31-(0)628498405

Information regarding the Data Management Committee, if established
N/A

Incidental Finding Reporting
An Incidental Finding is a finding concerning an individual research participant that has potential health or reproductive importance and is discovered in the course of conducting research but is beyond the aims of the study.

All incidental findings will be documented in a timely manner throughout the study. The report Incidental Finding Report Form (see ICBE SP) will be in the receipt of Philips Research Q&R Office within 7 business days of when the researcher first learns about the finding.

15 INVESTIGATOR BROCHURE (IB)

For this study an Investigator Brochure is provided to the investigator. Acceptance of this document will be documented in writing by the respective investigator.

16 VULNERABLE POPULATION

According to ISO 14155, a vulnerable subject is an individual whose willingness to volunteer in a clinical study could be unduly influenced by the expectation, whether justified or not, of benefits associated with participation or of retaliatory response from senior members of a hierarchy in case of refusal to participate.

☒ This study includes live human participants from non-vulnerable populations

☐ Children or viable neonate (birth to age 28 days)

☐ Cognitively impaired

☐ Pregnant women (except USA)

☐ Fetuses

☐ Neonates of uncertain viability or nonviable

☐ Prisoners

☐ Poor/uninsured

☒ Educationally disadvantaged (limited education, e.g., high school drop out)

☒ Students (including interns, residents, fellows)

☒ Minorities (includes migrants, refugees)

☒ Elderly (over age 65 years)

☐ Terminally ill

☒ Other (check box and specifically discuss below)

In this study adult patients will participate including patients which are educationally disadvantaged students, minorities and older than 65 years only in case they are able to understand the study procedures and are able to provide informed consent themselves and none of the exclusion and all inclusion criteria are fulfilled. Therefore, these are not considered as vulnerable population for this study.

To minimize the potential dependency of the patient to the investigators, the investigator informing on the study and collecting consent will not be the same as the one performing the surgery.

17 SUSPENSION OR PREMATURE TERMINATION

In case the study is suspended or ended prematurely, the investigator will notify the accredited METC and the ICBE a well as the RA about the reasons for the suspension or premature termination.

The principal investigator or authorized designee shall inform the subjects of the reasons for resumption.

The following criteria and arrangements for suspension or premature termination of the whole clinical investigation or of the clinical investigation in one or more investigation sites apply:

- Non-compliance to obtain patient informed consent
- Non-compliance to the inclusion/exclusion criteria
- Failure to follow patients per scheduled visits
- Failure to submit data in a timely manner
- METC or RA suspension of the trial or of the center

In case of suspension or premature termination of the clinical investigation, the following requirements apply for subject follow-up.

- Sponsor will promptly inform the clinical investigators of the termination or suspension together with the reasons why this decision was taken. They must also inform the regulatory authority(ies) (as stated by the applicable regulatory requirements).
- The EC will be promptly informed and provided with the reasons(s) for termination or suspension by the sponsor or by the clinical investigator.
- The investigator will promptly inform the patients and their personal physicians and assure appropriate therapy and follow-up for the patients.
- In case of early termination the investigator agreement will be terminated.

If the investigator (or METC) terminates or suspends the investigation without prior agreement of the Sponsor:

- The investigator will promptly inform Sponsor and the METC, and provide a detailed written explanation of the termination or suspension.
- The investigator will inform the institution (as stated by applicable regulatory requirements).
- The investigator will promptly inform the patients and their personal physicians and assure appropriate therapy and follow-up for the patients.
- The sponsor will inform the regulatory authority(ies) (as stated by applicable regulatory requirements).

18 PUBLICATION POLICY

It is planned to register this study in a public database like clinical trials.gov or Nederland trial register. All manuscripts, abstracts, or other presentations will be reviewed by the Sponsor prior to release. The detailed publication strategy is described in the eMTIC framework contract and the applicable Exhibit specific for this study between the Sponsor and the clinical site.

Authorship and attritubion will comply with ICJME guidelines, and privacy of participants will be safeguarded.

19 BIBLIOGRAPHY

[1] O. S. Ahmed, A. C. Rogers, J. C. Bolger, A. Mastrosimone, and W. B. Robb, "Meta-Analysis of Enhanced Recovery Protocols in Bariatric Surgery," J. Gastrointest. Surg., vol. 22, no. 6, pp. 964–972, 2018.

[2] A. Surve et al., "Does the future of laparoscopic sleeve gastrectomy lie in the outpatient surgery center? A retrospective study of the safety of 3162 outpatient sleeve gastrectomies," Surg. Obes. Relat. Dis., vol. 14, no. 10, pp. 1442–1447, 2018.

[3] M. C. Leepalao, D. Arredondo, F. Speights, and T. D. Duncan, "Same-day discharge on laparoscopic Roux-en-Y gastric bypass patients: an outcomes review," Surg. Endosc., no. 0123456789, pp. 7–10, 2019.

[4] HealthDot_accuracy_white_paper_interim_2_June2020

[5] Y. Q. M. Poelemeijer et al., "Textbook Outcome: an Ordered Composite Measure for Quality of Bariatric Surgery", Obes Surg. 2019 Apr;29(4):1287-1294. doi: 10.1007/s11695-018-03642-1.

- [End of document]
