# Supplementary material for: Telemonitoring for perioperative care of outpatient bariatric surgery: Preference-based randomized clinical trial
Source: PLoS One. 2023 Feb 22;18(2):e0281992. doi: 10.1371/journal.pone.0281992 (PMC9946229; doi:10.1371/journal.pone.0281992)
Supplement: Attachment — Submitted filename: Response to Reviewers.docx [file pone.0281992.s004.docx]

Dear Editors

Thank you for the opportunity to resubmit our manuscript. The comments of the reviewers were useful and have been addressed to accordingly, see below. Looking forward to hear from you.

Sincerely yours,

Lisa van Ede

On behalf of my co-authors.

Responses to the reviewers;

Reviewer #1

**Please edit the first line of the introduction paragraph for it to make better grammatical sense. Other than that no issues or concerns identified.** Thank you for this comment, we have altered this sentence.

Reviewer #2:

**Line 130-133 The description is not clear here. Those events mean “a perfect convalescent”?** Indeed, a perfect convalescent, outcome as hoped and described in a textbook, we clarified this as a composite measure of clinical process indicators. Textbook Outcome is realized for those who reached all desired short-term health indicators.

**Line 144 what test was used for the sample size calculation?** Sample size was calculated using an uncorrected chi-square statistic. This is added in the manuscript.

**Line 153: Which non-parametric test was used? Need to clarify which outcome was compared using t-test and which was compared using the non-parametric test.** The non-parametric test that was used was a Mann-Whitey U test. This is added to the manuscript. Also, we clarified which test was used for what outcome.

**Line 155: It is not appropriate to use two -sided p value for the non-inferiority trial.** Thank you for this comment, it is correct indeed that the results should be presented as one-sided p-values. The p-values in the first manuscript were results of chi-square tests. Now, these p-values were changed for p-value for non-inferiority testing using the Wald Method which is more correct. The numbers are altered in the abstract, main text and table 2.

1. Wellek S. Statistical methods for the analysis of two-arm non-inferiority trials with binary outcomes. Biom J. 2005 Feb;47(1):48-61; discussion 99-107.

**Line 152 & 156 what group means were mentioned here? Specifications of** group means of patients characteristics have been clarified.

**Line 178 the result of primary outcome comparison is not clearly written for a non-inferiority test.** The results in the paragraph for primary outcomes are rewritten using examples of existing literature and source 2

2. Piaggio G, Elbourne DR, Pocock SJ, Evans SJ, Altman DG; CONSORT Group. Reporting of noninferiority and equivalence randomized trials: extension of the CONSORT 2010 statement. JAMA. 2012 Dec 26;308(24):2594-604.

**Line 181. What are the 95% confidence intervals for event rates? One sided or two-sided. The confidence interval of event rate should be reported in the text and in table 2.** The 95% CI was only calculated on the combined outcome measure because the individual event rates were too low. The result of the individual events would not be reliable as the primary outcome was a combined measure, in accordance with advice of consulted hospital's methodologist. For this reason, also the p-values behind the event rates were removed from table 2. The 95% CI of the combined textbook outcome is presented 2-sided. This information is added to the methodology section.

**Line 179 and table 2: What is the purpose to use risk ratio?** The risk ratio was assessed and presented conform the consort guidelines for non-inferiority trials with binary outcomes (see link below). According to the guidelines, also the risk difference is added to the method and result section. *http://www.consort-statement.org/checklists/view/650-non-inferiority-and-equivalence-trials/827-binary-outcomes*

**The estimated risk ratio does not seem correct. Did you use odds ratio?** Correct, presented RR had to be adjusted. As a result also the Confidence Interval enlarged for which discussion and conclusion had to be adjusted.

**Line 180 & Table 2: what are the sample sizes for ITT and per-protocol? Is there any imputation used? If no data is missing (as specified in the title), what is the difference between the two?** It is correct that no data was missing, this was not a distinctive in analysis. Per-protocol analysis focused on those who were actually discharged the same day. In the ITT approach also those who did not succeed to leave the same day were included. Additional info has been added to the method section.

**Table 2. Add total N of each group. Omit risk ratio and add 95% CI for event rate. For mortality and etc., report N (%).** The table is adjusted except for 95% CI for event rates. The 95% CI was only calculated on the combined outcome measure because the individual event rates were too low. The result of the individual events would not be reliable as the primary outcome was a combined measure, in accordance with advice of consulted hospital's methodologist. Also for this reason the p-values behind event rates are removed.

**Line 224 all patients were prescribed. Why n=10 and 9 in RM and SC respectively?** Correct, it was a typo error, altered into ‘In addition, patients from both groups were prescribed the same amount of opioids after being discharged (10/102 (11%) versus 9/100 (9%) in SC group p=0.82).’

**Table 3 is confusing.** The layout is upgraded for more clarification.

**Table 4 add (%)** Adjusted
